# Integrating Building Information Modeling and Health and Safety for Onsite Construction

**DOI:** 10.1016/j.shaw.2014.10.002

**Published:** 2014-10-18

**Authors:** Abdulkadir Ganah, Godfaurd A. John

**Affiliations:** The Grenfell-Baines School of Architecture, Construction and Environment, University of Central Lancashire, Preston, UK

**Keywords:** building information modeling, communication, construction, health, safety

## Abstract

**Background:**

Health and safety (H&S) on a construction site can either make or break a contractor, if not properly managed. The usage of Building Information Modeling (BIM) for H&S on construction execution has the potential to augment practitioner understanding of their sites, and by so doing reduce the probability of accidents. This research explores BIM usage within the construction industry in relation to H&S communication.

**Methods:**

In addition to an extensive literature review, a questionnaire survey was conducted to gather information on the embedment of H&S planning with the BIM environment for site practitioners.

**Results:**

The analysis of responses indicated that BIM will enhance the current approach of H&S planning for construction site personnel.

**Conclusion:**

From the survey, toolbox talk will have to be integrated with the BIM environment, because it is the predominantly used procedure for enhancing H&S issues within construction sites. The advantage is that personnel can visually understand H&S issues as work progresses during the toolbox talk onsite.

## 1 Introduction

The Plan for Growth by the Government in the UK, published alongside Budget 2011, emphasized the significance of an efficient construction industry in the country to the economy. Construction accounts for about 7% of the gross domestic product—or £110 bn of expenditure per year—with approximately 40% of this being in the public sector, with Central Government being the biggest customer of the construction industry [1].

The construction industry is well known as one of the most dangerous in which to work [2]. Despite the fact that the UK construction sector only accounts for approximately 5% of employees in Britain, 27% of all reported occupational fatalities and 10% of major injuries are from the construction industry, as reported by the Health and Safety Executive (HSE), the body that manages health and safety (H&S) in all sectors of the UK [3].

Most of the recommendations proposed by Sir John Egan in “Rethinking Construction” in 1998 have now been implemented by the construction industry. However, most large construction firms have made a tremendous effort to achieve the recommendation of a 20% reduction in accidents. Although problems exist with construction firms that make up the bulk of the industry, the awareness remains among practitioners and academics in finding innovative solutions to address most of what was said in the Egan report. The HSE has also made progress in the areas of H&S regulations, guidelines, and approved codes of conduct, as well as creating H&S awareness among construction practitioners.

As construction projects increase in complexity, alternative modern methods of construction and design increase in popularity [4]. These new, complex construction projects require new forms of innovation in design and methods of construction. To address this matter, Suermann [5] pointed out that building information modeling (BIM) can be used by designers, construction managers, and contractors to accomplish tasks more efficiently than ever before and pave the way for future construction professionals.

Hence, with the development of BIM and the life cycle realization of the project in one holistic environment, most of the H&S information can be created directly in this single environment. These are some of the advantages that this article intends to explore in relation to the use and application of the integrated approach within the four-dimensional (4D) modeling for the benefits of most site workers.

The research questions for this article will only address the issues of H&S on a construction site, as well as investigating the position of practitioners' perception with respect to site operatives on BIM usage for H&S on-site construction.

In the wider literature there are many definitions of what BIM is, and in many ways it depends on the point of view of who looks at it or what is sought to gain from the approach [6–8]. However, in the UK, the Construction Project Information Committee (CPIC) has defined BIM as: “… digital representation of physical and functional characteristics of a facility creating a shared knowledge resource for information about it forming a reliable basis for decisions during its life cycle, from earliest conception to demolition” [9]. Hence, for this article we view BIM as a “catalyst” for site practitioners and operatives to improve and enhance their safety concerns and their understanding of the dynamic site environment, as they carry out work activities.

The goal of this article is to answer the question: “How can BIM enhance the continuous improvement of Health and Safety on construction site?”.

## 2 Materials and methods

The methodology used in this article followed a traditional literature review approach to understand the development of BIM technology up to its current status. The approach, however, concentrates on the thematic analysis of literature in which relevant themes affecting the application of BIM onsite are investigated. For each research theme, the classic and contemporary theory that underpins each is first investigated, and then the literature review that is undertaken is couched within the theoretical underpinning framework. This work starts with the literature review on communication theories with particular references to H&S on construction sites. An intensive literature search was conducted on the following main themes: BIM innovation; H&S implementation; and communication issues on construction sites. The three themes were then brought together to understand important factors as well as barriers of H&S communication on construction site. These themes were further explored and validated in a questionnaire survey sent to practitioners.

A questionnaire was used to facilitate the collection of information from construction firms. It covered issues relevant to H&S performance as well as those related to BIM. The allocation of items to domains was a matter of judgment but was guided by discussions with H&S practitioners within the built environment and the HSE itself, as well as by previous surveys carried out on this subject. To a certain extent, the different statements used in developing the questionnaire were based on scales that had been previously used by researchers [10–16].

All the survey questions were measured through a Likert-type response format. Properties relating to each of the survey questions were used in the form of statements to measure personnel's understanding of the topic under investigation. Participants were asked to endorse the statements using a 5-point Likert-type scale from 1 ‘‘strongly disagree’’ to 5 ‘‘strongly agree’’.

The draft questionnaire was reviewed by five practitioners, which gave the questions a better content validity, prior to the distribution of the survey questionnaires to the chosen organizations. The self-administered questionnaires were distributed to a sample of 200 construction practitioners in the UK in mid-2012. The sample was randomly selected from the top 1,000 practitioners based on their annual turnover. Altogether, 46 questionnaires were returned and analyzed, giving a response rate of 23%, which is an acceptable return for a questionnaire survey [17].

Prior to sending the questionnaire out by postal mail to senior personnel, the data reliability and validity were tested. Data reliability is associated with the data source, data collection instrument, and the quality of the communicated questionnaire, as well as the identification of the position held by the respondent [18,19]. Based on the profile of the respondents, the direct mailing to individuals in organizations seemed to have achieved its objective of reaching those who were closely involved with delivering construction projects. Posting to organizations in different regions of the UK minimized the duplication of selected projects. All 46 respondents provided their business details, which revealed that all held senior positions within their organizations and have influence in the management of H&S.

### 2.1 Communication in construction

There are several theories of communication [20–23]. In this article a perspective of these theories is taken that aligns itself with the practices of the construction industry. The theories of communication more suited to construction are the linear approach, the interactional approach, and the transactional approach. The linear approach suggests that the person is only a receiver or a sender. The interactional model of communication emphasizes the two-way communication process between the communicators. In other words, communication goes in two directions. This circular process suggests that communication is ongoing. The transactional approach underscores the simultaneous sending and receiving of messages in a communicative episode. To say that communication is transactional means that the process is cooperative; sender and receiver are mutually responsible for the effect and the effectiveness of communication. In the linear model of communication, meaning is sent from one person to another. In the interactional model, meaning is achieved through feedback of sender and receiver. In the transactional model, people build shared meaning [21].

Communication also includes both the communication medium and the core knowledge which form the basis for mutual understanding of team participants. Bennett [24] has stated that there are two categories of interaction that match the basic characteristics of teams. The first is concerned with the communication of information. Information should be first translated into text or graphics that the other team is likely to understand. These texts or graphics need to be communicated to the other team through a communication medium [25–27]. The second category of interaction is the one concerned with work organization. Clear organization of work allows the work of teams to fit together. In other words, teams should coordinate their actions.

In the construction industry, site teams and other participants in construction projects communicate using traditional methods such as face-to-face meetings, paper-based drawings, schedules, written statements. The construction industry displays some inertia in changing its methods of communicating and innovating through adopting new technology. The use of telecommunication systems such as facsimile, e-mail, and mobile phones has improved the communication in respect of speed, but it has not influenced the efficiency of the process or the quality of information exchange [28,29], especially with respect to H&S of all the workers onsite. In recent times there have been great leaps in technology for onsite communication, with the introduction of wireless internet, personal digital assistant (PDA) systems, internet protocol communications, and computer-aided design, as well as BIM technology and innovations. The knowledge about such new technologies is prevalent within the industry but tends to concentrate in its upstream end, that is the design and consultancy area, as well as in educational institutions. This knowledge needs to be translated to the downstream end of the industry, that is, onsite activities through subcontractors. The core knowledge to be communicated within the different participants is known, but needs to be translated into the new medium for communicating in an effective and efficient manner.

Management is expected to use a variety of formal and informal means to promote and communicate its commitment to safety [30,31]. Some authors claim that both management communication and employee feedback are critical for safety improvements and reporting near misses as well as unsafe conditions and practices [32]. Most current H&S communication on construction sites tends to be one-directional without feedback from the operatives or recording of them carrying out their activities.

### 2.2 H&S management: planning and communication

The practice of H&S planning and management in construction can be summarized in the following areas [33,34]: (1) safety legislation, regulation, standards and guidelines; (2) appointment of construction and design management (CDM) coordinator by the client; (3) designers' H&S considerations upstream in the creation of the artefacts and its associated hazards and risks consequences. (4) management of these H&S risks during construction for site personnel, in particular writing-up method statements for work activities; and (5) development of an H&S plan and the creation and development of the H&S file through the life cycle of the project.

H&S management is, therefore, part of the wider planning and management process of the construction project that was developed in the upstream end of the product life cycle, to be implemented at the downstream end.

The design team occupies a unique position in the construction planning process, not only through design *per se* but also through the associated role of professional adviser to the client. Design insight and technical advances are unfortunately built on the foundations of design failure. Although a designer occupies a central position in H&S management within the project process, he/she is also only one of three or five duty holders, depending on the status of the project.

Preconstruction information is associated with all projects and shall represent an information stream toward which all duty holders contribute and from which they all receive relevant information at the appropriate time—that feeds forward and feedback. It is triggered by the client on all projects. The designer is entitled to base his level of communication on the premise that contractors are competent and hence there is no need to provide a focus on issues which a competent contractor would normally expect to deal with. Concise, focused documentation is a vital tool in this communication linkage.

The project risk register (H&S), which is one such document, is a collection of the significant and principal H&S issues, collated from the project team members' input into one central document, with ownership, resolution times, and outcomes all recorded. Its use enables the team to focus on all of the actions needed and, by review and suitable document management, on the developing strategies and methodologies required [35].

The design management process can fail through ineffective communication, lack of ownership, team fragmentation, resource limitation, incompetence and inappropriate level of awareness, risk management failure, complacency, reactive responses, infrastructure deficiencies, confusion, and unrealistic timescales [35]. The designers' focus on competent contractor(s) to some extent is a fallacy, as not all the subcontractors onsite would exhibit the competency attributes level required and hence raise H&S concerns.

Construction sites undergo dynamic change in ways that fixed industrial facilities do not; work teams (i.e., work gangs) are transient, the physical structure and spaces change, as well as the environmental conditions (weather). Another difference is that in construction, workers of one team are frequently exposed to dangers posed by the workers of other, unrelated teams. Performing risk analysis prior to any activity at any time is essential but difficult, even if the same activity is performed repeatedly, because the site conditions change through time. This demands more effort than most contractors or workers are willing to invest, and therefore safety management in construction sites commonly suffers from low level of efficiency, with effective risk analysis performed only rarely [36,37]. In the absence of an efficient and effective way of predicting peak risk levels, safety management on construction sites is performed at a constant level of effort, focusing on provision and use of personal safety equipment, training, accident and near-miss investigations, and taking steps to fulfil regulatory requirements [37]. Hence, as design and construction information become increasingly digitally communicated, the Architecture, Engineering, and Construction (AEC) sector needs to invest more in capabilities concomitant with both technology and skills of using such technology to help improve H&S on construction sites [38].

### 2.3 The technology

Currently, each construction project is complex and dynamic, which makes construction planning, design, site, and management complex and difficult [39]. Professional work has become digitally communicated and distributed; the making of buildings and infrastructure involves substantial and local physical labor. Yet this work is not unchanged by the digital economy. In any particular project, safe practices draw both on standardized regulations and tools, and are locally emergent. Such new tools and practices will instead be judged by the extent to which they foster consideration of safety through the kind of “mindful” actions that challenge assumptions, check and validate proposed solutions, and make sense of and respond to unexpected situations [40].

To bridge some of the gaps in the aforementioned reasons for onsite communication, it is expedient to research into the use of current digital communication with the intention to garner information on how these digital communication will enhance the onsite processes. By so doing, more of the digital communication will be useful in conveying new or improved methods of working on the construction site. BIM is considered as one of the new tools that will bridge this chasm.

BIM is widely viewed as an enabling technology with the potential for improving communication between stakeholders, improving the quality of information available for decision making, improving the quality of services delivered, and reducing time and cost at every stage in the life cycle of a building [41]. One of the key advantages of BIM over two-dimensional (2D)and 3D computer-assisted design (CAD) is that BIM represents and manages not just the graphics but also information—information that allows the automatic generation of drawings and reports, design analysis, schedule simulation, facilities management, etc., ultimately enabling the building team to make better-informed decisions. Thus the increasing use of BIM in AEC sector may help in changing the way safety can be approached [42].

The planned sequence of work is usually part of BIM, which can be used later to produce animations of the construction process of a building over time; therefore, showing how the work onsite should be carried out according to contractual responsibilities. In addition, BIM has the potential to be used beyond the design stage to include the construction and operation of a virtual building that parallels the real building [43]. Thus, the technology can prove crucial to the success of a project by effectively controlling the construction schedule, budget, quality, and reducing risks [44], through time-controlled realistic simulation.

BIM technology has the potential to be used in safety planning procedures, particularly those related to tasks on construction sites. Four-dimensional modeling tools can be used to link a project's scope in 3D with the construction schedule to simulate the construction process graphically. Construction tasks onsite can be modeled in a 4D CAD production model, in which the model produced by designers is used as the starting point. Previous studies have found that certain sets of movement characteristics for construction facilities, such as tower crane movement and movement of construction vehicles, is possible [45,46]. This may enable the system to simulate the construction more realistically [39].

Most of the research in BIM with respect to the AEC industry has dealt with preconstruction phases [47], a recent exception being Davies and Harty [29], dealing with “Site BIM”. Harty et al. [48] investigated the use of BIM to assess the access adequacy for installing new services and performing H&S assessment—looking for trip hazards. Zhang et al. [42] developed an automated hazard identification and correction platform during design and construction planning stages. Larsen and Whyte [38] studied the effect of designers' work on the safety of operatives on the construction site. These projects did not investigate how H&S onsite during construction can be assessed and addressed. The utilization of 4D BIM technology may improve occupational safety by connecting the safety issues more closely. However, the uptake of this technology within the construction industry is partial and fragmented [48,49].

The literature review shows that, currently, BIM usage is confined mostly to the design and planning stages of the project, with very little of it being used in the construction phase in relation to H&S through hazard perception. However, the construction phase is where the bulk of accidents and H&S occurrences are recorded. Also, it is in the construction phase during which personnel induction is ongoing, due to the high turnover rate of personnel in the construction industry. Concentration of contracting firms (i.e., medium and small firms and subcontractors) is predominant within the construction phase. New methods are needed to help alleviate some of these problems that may be contributing factors in appreciating H&S management proactively.

There are three basic benefits of a BIM-based methodology, namely: 3D simulation over 2D representation; accuracy over estimation; and efficiency over redundancy. Although 2D is merely an abstract representation of a building form in plans, elevations and sections, BIM allows 3D simulation of the building and components.

In order to understand how practitioners are using and interacting with the current digital communication methods, a questionnaire survey was conducted which constituted the following elements: (1) current communication methods on construction sites; (2) BIM current status; (3) H&S management through the H&S file; and (4) prioritizing H&S documentation according to practitioners' preferences.

## 3 Results

### 3.1 H&S: practitioner perspective and views

The results from the questionnaire survey analysis are divided into the following areas: the characteristics of the organizations, the perceptions of practitioners, and the H&S methods on construction sites.

### 3.2 Characteristics of the respondents and organizations

Table 1 includes the demographic information provided by the respondents about the type of work performed by the organization, company size, and years of experience. The design firms that took part in the survey appear to be large organizations involved mostly in general building and civil engineering projects, and active in local, national, and international markets.

More than 50% of the organizations have >250 employees and only 2.2% of the respondents have <50 employees in their organization. Contractors were the largest group that responded to the survey, with a percentage of 61%. Seventeen percent of the respondents were multidisciplinary in nature and the rest were mainly from a diverse engineering background, ranging from building services engineers to civil engineers.

Approximately 94% of respondents have over a decade of working in their profession. This gave a good indication of the group that was targeted for the questionnaire survey.

### 3.3 Current perception of practitioners to BIM usage

From the analysis of the statistics, the current perception of practitioners in the upstream end of the construction life cycle is that they have embraced the emerging BIM technology and its usage (Table 2), whereas contractors appeared not to have embraced the technology that much. The former believe that it will benefit them profusely and that savings can be made efficiently. Also, comments made by practitioners show that they are a bit skeptical about using BIM for H&S, as well as induction training onsite.

### 3.4 Current H&S communication methods on construction site

From the analysis of results as presented in Table 3, expressed as percentages from practitioners' responses, it appears that toolbox meetings, followed by workshops/seminars, and the HSE guides are regarded as the current best forms of communication to site operatives. Currently, onsite communications do not use BIM and other visualization tools; it is not a common practice. It may appear that BIM currently is not so well known and therefore scored the least. The reasons for this may be that contractors onsite are not yet familiar with the BIM technology and the innovation and value-added possibilities it will bring. In addition, fragmentation of the practitioners does not lend itself to effective and efficient communication pathways. Practitioners from different disciplines are not comfortable about sharing their knowledge on this new innovation. Therefore, the diffusion of the technology is slow to assimilate to those downstream; that is, the subcontractors in the construction process.

Although meetings (i.e., toolbox meetings) are recorded, they are not captured in vitalization format, such as feedback in other forms of communication (i.e., body language) can be reported back to site operatives. Also, some aspects of communication that require visualization mainly revert back to 2D methods of communication, which does not bring out successfully the safety elements required, especially in variation order.

In the survey, a key question was asked: “To what extent do you think that it would be feasible for a single guide, such as in “H&S” to adequately cover the main H&S issues on the construction site?”. From Table 4, we can see that 66% of practitioners favor the introduction of a single H&S guide that will be used onsite; some of the respondents commented that the guide will be even better if it can incorporate visualization in the way it is presented. However, 27% think that this is probably not feasible, which represents a substantial number. We should not forget that H&S itself is self-regulated through a single organization, the HSE; since its inception, it has consistently been driving construction accidents down significantly. It is, therefore, feasible that a single guide or point of entry will augment the way H&S will be practiced onsite, if fully controlled by the regulating body. This is where BIM technology comes into play.

## 4 Discussion

In construction, it is apparent that most practitioners within the industry still believe that the H&S file is the single most important repository within the UK construction project, especially at the implementation phase for all H&S [35]. Further informal discussion with practitioners revealed that BIM will be a duplicate information resource and that it is not necessary.

However, although some of these practitioners are adamant about this duplication, they did not take into consideration that the safety file cannot show any form of visualization at a specific point in time. With BIM, construction stages that are linked through schedules can be extracted and explained in real time, which gives a better understanding for all operatives on the construction site, as issues can be visualized.

A toolbox meeting, the dominant mode of communication on a construction site, is only a two-directional communication approach that is not conversational and transactional in nature. Within BIM, the visual nature of the environment will create conversation about what is expected onsite and it will be transactional, as site operatives whose H&S is affected, will create a transactional understanding of the work activity to be carried out. If they are not sure about issues of safety affecting them, then they can always refer back to the BIM environment to augment their understanding. In a toolbox meeting, the meeting space for all practitioners will be in one location at any instant in time. However, with BIM, merely synchronization of the software environment is required. The meeting can be held virtually, with designers in different locations at different times.

Moreover, because H&S issues and characteristics are different attributes connected to activities within the BIM environment, accessing issues of H&S can correctly be tracked back to all original objects that they applied during the product development. However, the H&S file is a database that has to be investigated like any other database, but not fully tied into specific work, task, or activities that have been performed.

By making H&S a property associated with each work activity or package within the BIM environment, changes, alterations, and improvements will be associated with that work activity or package (i.e., considered as a BIM object), into which a sound knowledge base is beginning to develop and less work is needed in understanding what the real issues are at that specific point in time.

From this small-scale survey conducted, though not conclusive, it is shown that practitioners are a bit hesitant; however, the idea of change in work process is rather the problem here, as people do not fully know the depth and breadth of BIM technology for the construction industry. Also, the survey shows that with good technological understanding, BIM will reveal issues that are not so apparent when other methods are used for H&S management. The uptake of BIM by the industry will happen despite such apprehension of practitioners, because government is the driver of the change in this area [1]. The time required to achieve this is not as certain, since each section of the industry is also looking at the cost of such an uptake in this technology.

According to these findings, there is a lack of awareness among contractors and some practitioners of BIM usage as well as its possible uptake in the near future. BIM offers a new way of communication which is not possible with the current tools used in communicating H&S information. This new way allows for information to be fed forward and backward.

If BIM usage enforced by the government is to meet the target date (2016), then a huge effort is required in the industry to drive that forward, through sensitization from workshops, seminars, and market dissemination.

In conclusion, new directions of research on construction site safety should focus on technologies that enable construction site operatives to share their knowledge and experience with designers, using the BIM potential to bring knowledge of the construction site into design, in which most of the cost committed to the project must have been realized. However, from the survey conducted, it would be expedient that we find a way of integrating toolbox talk and BIM for the construction environment, as toolbox talk came out as the dominant issue that will still be essential for the foreseeable future on construction sites.

One area that is required for the uptake of BIM in the industry is to prepare contractors and site managers, as well as those close to the cutting-edge technology, on how to augment change and what is expected when change is introduced. Without that, it is rather difficult to claim that people will be willing to use such a best practice in the near future. Another area of interest with regard to those most hesitant about BIM is the fact that information and communications technology software changes or needs upgrading every 3 years, which adds a lot of cost to the initial value of the technology. Lastly, with new innovation comes new roles and responsibilities, as well as working patterns. In terms of the working pattern, BIM need to be integrated seamlessly with other packages like project management programming software. Currently, the industry does not have sufficient qualified professionals in large numbers to be able to meet all the new demands that BIM will bring. Therefore, one strategic area of the development in the construction industry is to increase the users and practitioners of BIM through concerted educational courses to meet projected demands.

## Conflicts of interest

The authors declare no conflicts of interest.

## Figures and Tables

**Table 1:** Demographic information of the respondents

Characteristics of respondents	%
Type of service provided	
Multidisciplinary	17.8
Project management	2.2
Civil engineers	2.2
Building services Engineers	2.2
Contractors	62.2
Others	13.3
Size of organization	
1–50 employees	2.2
51–250 employees	41.3
>250 employees	56.5
Number of years of experience	
6–10	4.4
>10	95.6

**Table 2:** Building information modeling usage

Response	%
Not at all	37.0
Rarely	26.1
Sometimes	21.7
Frequently	13.0
Always	2.2
Total	100.0

**Table 3:** Current onsite health and safety communication medium

Method	Most likely	Next most likely	3rd most likely	4th most likely	5th most likely	6th most likely	7th most likely	8th most likely	9th most likely
(1) Safety toolbox meetings	77	12	—	9	—	—	—	—	2
(2) Health and Safety Executive guides	2	28	19	18	21	7	5	—	—
(3) Building information modeling	—	2	2	10	9	5	17	17	38
(4) E-Learning	—	—	5	—	12	20	29	27	7
(5) Simulation onsite	5	17	38	5	9	5	9	7	5
(6) Workshops/seminars	17	31	19	24	2	3	—	2	2
(7) DVD	7	20	12	15	22	12	5	5	2
(8) Downloaded files	—	5	7	19	19	26	5	12	7
(9) Distance learning	—	—	2	2	7	14	23	23	28

**Table 4:** Respondents' view on a single health and safety guide

Response	%
Very feasible	31.8
Feasible	34.2
Unsure	6.8
Probably not feasible	22.7
Definitely not feasible	4.5
Total	100.0
